# Metabolomic differential analysis of interspecific interactions among white rot fungi *Trametes versicolor*, *Dichomitus squalens* and *Pleurotus ostreatus*

**DOI:** 10.1038/s41598-017-05669-3

**Published:** 2017-07-13

**Authors:** Feng Luo, Zixuan Zhong, Li Liu, Yasuo Igarashi, Deti Xie, Nannan Li

**Affiliations:** Research Center of Bioenergy and Bioremediation, College of Resources and Environment, Southwest University, Beibei, Chongqing 400715 People’s Republic of China

## Abstract

Interspecific fungal antagonism occurred commonly in the interaction zone of different white rot fungi. This competitive interaction could markedly influence the metabolic pathway of intracellular metabolites, which was associated with the fungal morphology change and growth restriction. So far, it remains unknown on intracellular metabolite regulation during fungal competitive interaction. Herein, we performed the metabolomic analysis of the *in vivo* metabolite changes during competitive interaction between each two of the three white rot fungi *Trametes versicolor*, *Pleurotus ostreatus* and *Dichomitus squalens* and identified differential metabolites in the interaction zone compared to each two isolates. Many metabolites in the carnitine, lipid, ethylene and trehalose metabolic pathways were significantly up-regulated. These metabolic pathways are all involved in defensive response to abiotic and/or biotic stressful condition.

## Introduction

Competition is the most common type of interaction occurring between white rot fungi^1–3^. When different fungal species confront with each other, direct combative interactions between mycelia to defend or compete for resources are always accompanied by changes of mycelial morphology and secretion of extracellular metabolites^1, 3, 4^. These metabolites have been identified including alcohols, aldehydes, ketones, terpenes and aromatic compounds, most of which are important in defensive response to interspecific antagonism in the interaction zones^5^. As it has been reported that oxidative stress can be induced during the interspecific interaction^6^, some oxidative enzymes were enhanced in the interaction zone to mediate the oxidative stress by removing reactive oxygen species (ROS), such as laccase and peroxidases^7, 8^, which also implied another extracellular signaling and metabolic pathway.

Metabolomics-based approach has been used extensively for understanding metabolites respond to environmental stimuli in fungi, bacteria and plants^9–14^. Metabolomics is increasingly being applied in identifying the target metabolites produced by fungi during stressful condition. Nowadays, some studies showed that the secreted metabolites in the interaction zone of two fungi species caused related mycelial morphological phenotypes, suggesting the changed metabolites could be also involved in the defense response against abiotic stress^15–20^. Some potentially novel metabolites belonging to natural products were induced and detected during co-culturing of fungi on solid media, and these metabolites were released in response to antagonistic interactions^17, 19, 21, 22^.

There was no report focusing on the differences of *in vivo* cellular metabolites, especially those serve as a composition of cell membrane and cell wall structure, which can affect the morphology of the cells during fungal antagonistic interaction. Until now, the intracellular metabolic regulation mechanism against stressful condition are still less characterized. It has been known that various pathways can be motivated to enhance the competitive potential of each species^1, 3^, which might involve signal molecules, growth inhibitors or toxins, and their by-products in this barrage zones^3, 16, 18^. Therefore, even though the two fungi confronting on the plate were different species, there may be common strategies in response to the interspecific interaction.

In order to study the differential metabolic response between competitive mycelial interaction zones and corresponding isolates, as well as to unravel the effects on the *in vivo* metabolic network, we performed the metabolomics analysis of interaction zone between each two of the three white rot fungi by ultra-performance liquid and/or gas chromatography coupled to mass spectrometry (UPLC–MS and GC–MS) based on metabolomics approach. The three white rot fungi *Trametes versicolor* (Tv), *Dichomitus squalens* (Ds) and *Pleurotus ostreatus* (Po) were chosen based on their significant interspecific laccase induction. The study is important to reveal differential levels of intracellular metabolites when fungal mycelia interact with each other, which can be applied into the further analysis of metabolite signaling research.

## Results

### Synergistically changed metabolites in all three interactions

**Figure 1:**
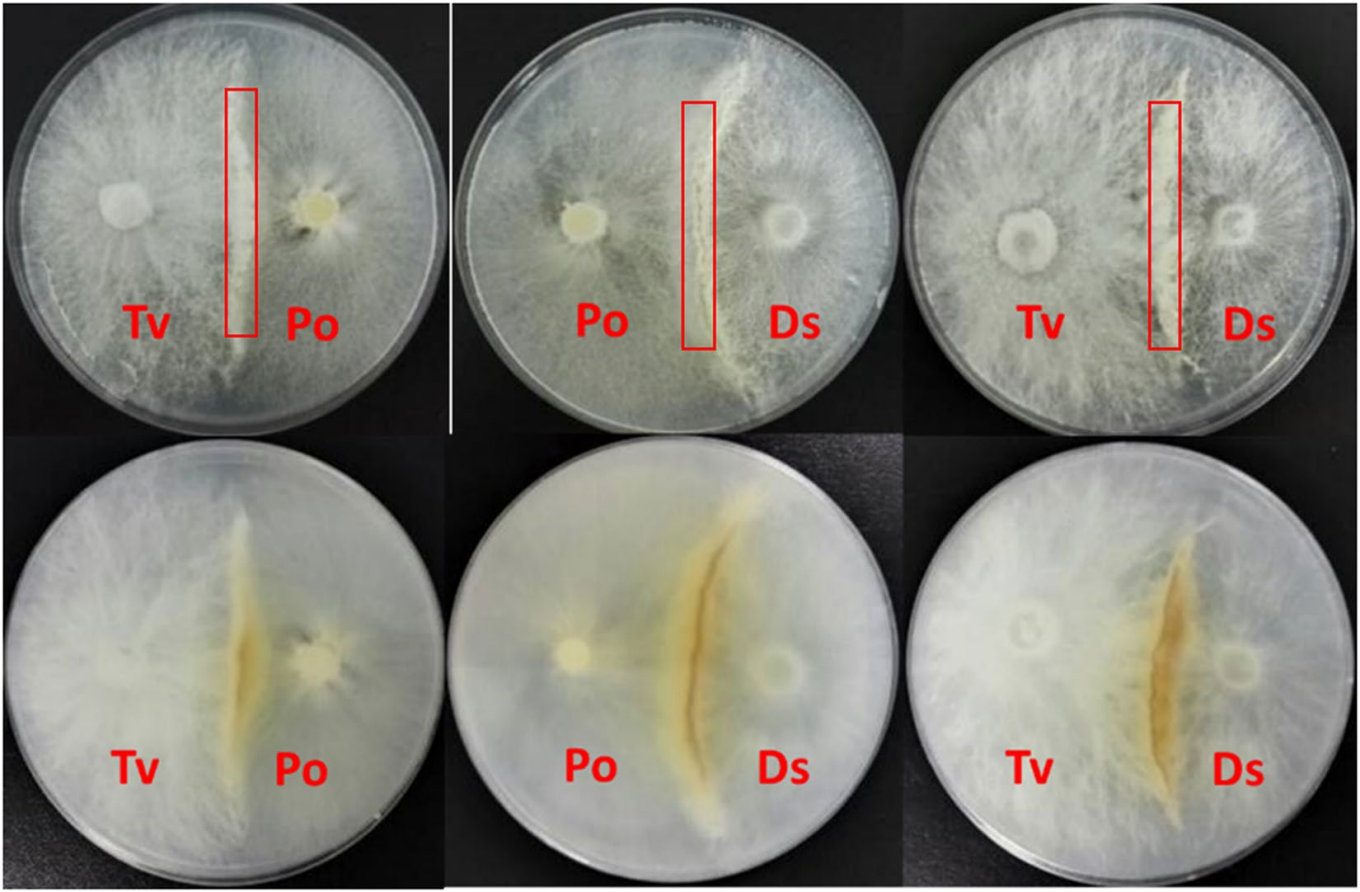
Paired-culture on Sc agar plate among Tv, Ds and Po.

The metabolomic dataset was comprised of total 279 compounds of known metabolites from the three white rot fungi isolates Tv, Ds and Po and their interaction zones (Abbreviated as TvDs, TvPo, PoDs; Fig. 1). The false discovery rate and statistical significance were calculated to represent the multiple comparisons between interaction zones and the relevant isolates (Supplementary Table S3). Moreover, the significances of the differential metabolites were confirmed by a pairwise comparison to the t-test. The total identified differential metabolites with intensity values were listed in Supplementary Table S1.

**Figure 2:**
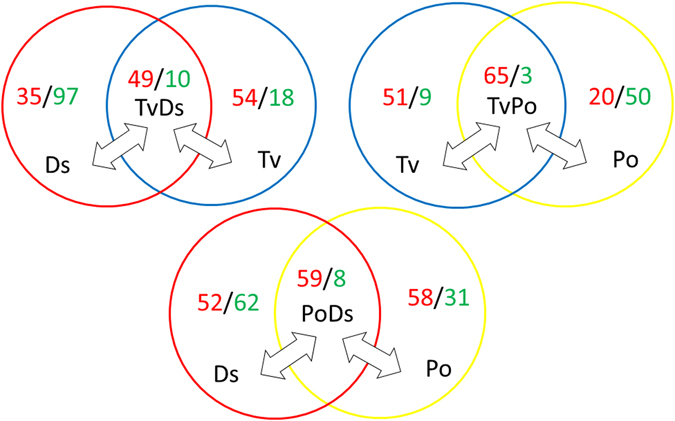
Significant changes of compounds in each interaction zone compare to the two isolates. Ratios as: up-regulated numbers/down-regulated numbers, p-value ≤ 0.05 was taken as significant.

The detected 279 compounds covered various metabolic pathways such as metabolic pathways of purine, pyrimidine, amino acid, central carbon, TCA, sugar, glyoxylate, alkaloid, photosynthetic, phenylpropanoid, and flavonoid metabolism. Based on the metabolomics data, we compared compounds which met the statistical criterion for significance (p-valve ≤ 0.05) in the three interaction zones of TvDs, TvPo and PoDs to their respective isolate zones of Tv, Ds and Po. The result showed that in most cases, there were more compounds in the interaction zone relatively increased, not decreased, comparing to the isolates. Only the Ds isolate relative to TvDs interaction zone had more increased compounds (Fig. 2). Moreover, over half of the increased compounds in the interaction zone were synergistically increased in all three interaction zones when compared to both isolates. For instance, 65 synergistically increased compounds and only 3 synergistically decreased compounds in TvPo comparing with isolates of Tv or Po(Fig. 2).

**Figure 3:**
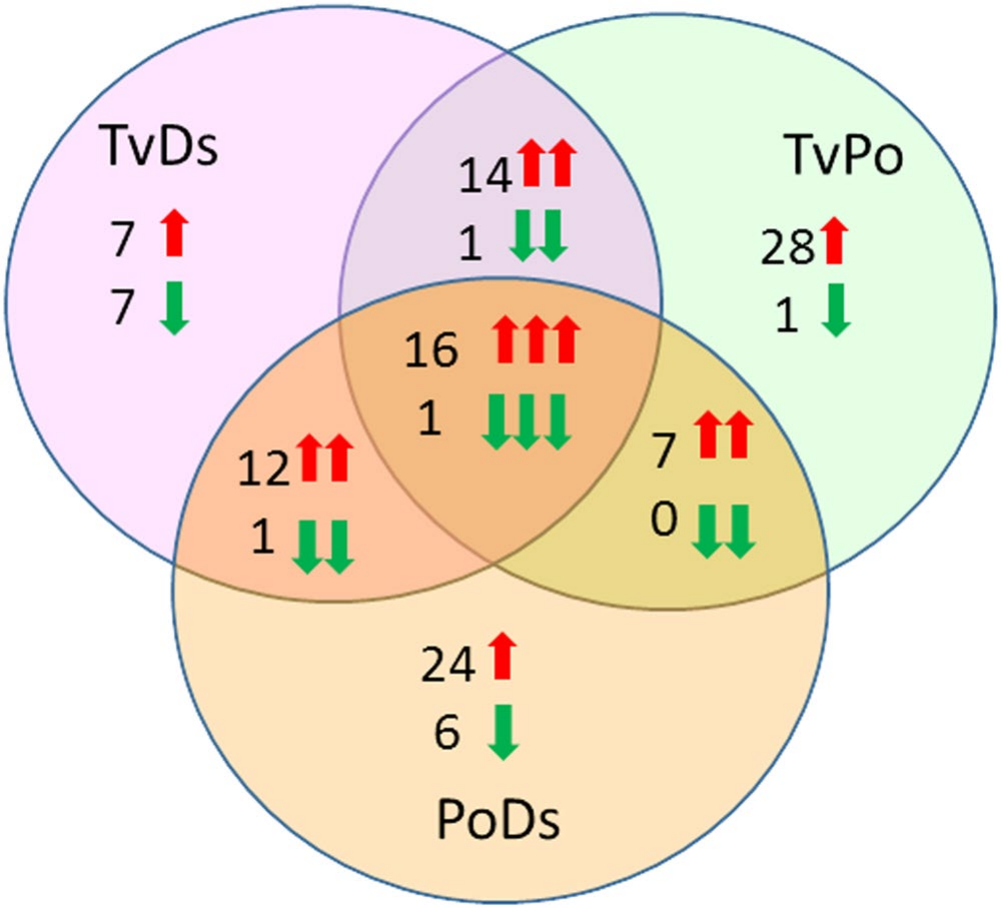
Summary of the pathway-level increase (red box) and decrease (green box) of metabolism in three interaction zones with significant data combined from synergistically changed metabolites compared to both isolates. Pathways in yellow and white fonts represent unique and shared pathways, respectively.

Interestingly, these synergistically changed metabolic pathways showed some similarities (Fig. 3). In the interaction zone of TvDs, the changed (increased) pathways were all shared with TvPo or PoDs, and the number of decreased pathways in TvDs was more than that in TvPo and PoDs. Four unique decreased pathways can be seen in TvDs, including ascorbate metabolism, fatty acid, dicarboxylate, glutamate family (alpha-ketoglutarate derived) and photorespiration pathways. In the interaction zone of TvPo, the number of increased pathways was more than that in TvDs and PoDs. Six increased pathways were unique in TvPo, including C5 branched dibasic acid metabolism, calvin cycle and pentose phosphate, CoA metabolism, free fatty acid, glutathione metabolism and photorespiration pathways. Only three increased pathways were unique in the interaction zone of PoDs, including benzenoids, Branched Chain Amino Acids (OAA derived) and oxylipins pathways. Otherwise, there were also three unique decreased pathways in PoDs, including free fatty acid, glycolysis and sucrose, glucose and fructose metabolism pathways (Fig. 3).

**Figure 4:**
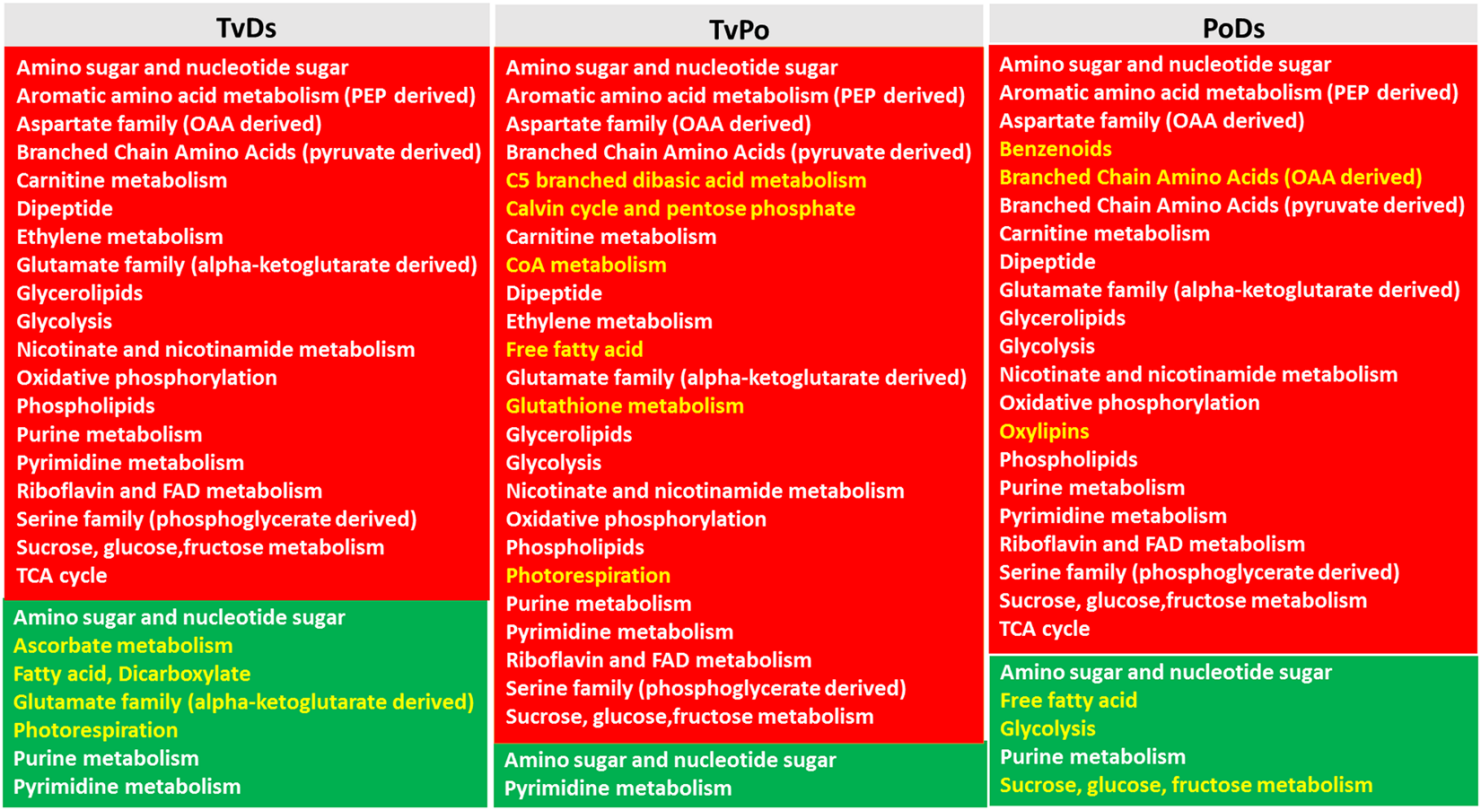
Synergistic changes in all three interaction zones relative to both isolates. Red arrow means increase, green arrow means decrease.

To analyze these significantly changed metabolites more globally, we also investigated them in all interaction zones among the three different white rot fungi (Fig. 4). We found 16 metabolites synergistically increased and only one synergistically decreased compounds in all interaction zones, the pathway classification and fold-change calculations for all 17 synergistic metabolites were shown in Table 1. According to the result, the aromatic amino acid tryptophan was one of the most strongly induced compounds in all three interactions. Notably, the fold change of TvPo and PoDs compared to Po isolate reached up to 119.94 and 71.73, respectively. The major commonality of other compounds was that most of the compounds were associated with catabolic processes, including branched chain amino acids metabolism, carnitine metabolism, glycerolipids metabolism and phospholipids metabolism. Two groups of compounds stood out as being overrepresented, they were group of various carnitine derivatives and another group of lysolipids and glycerolipid catabolites.

**Table 1:** Statistically significant metabolites in all interactions.

Super Pathway	Sub Pathway	Biochemical Name	Fold Change					
			TvDs/Tv	TvDs/Ds	TvPo/Tv	TvPo/Po	PoDs/Ds	PoDs/Po
Amino acid	Branched Chain Amino Acids (pyruvate derived)	2-methylbutyrylcarnitine (C5)	3.6	2.61	3.27	2.05	3.8	3.28
	Branched Chain Amino Acids (pyruvate derived)	isobutyrylcarnitine	14.57	4.6	12.13	1.82	7.93	3.76
	Branched Chain Amino Acids (pyruvate derived)	propionylcarnitine	2.64	3.91	5.24	2.05	9.73	2.58
	Aromatic amino acid metabolism (PEP derived)	tryptophan	6.29	11.04	8.18	119.94	8.58	71.73
Carbohydrate	Glycolysis	Isobar: hexose diphosphates	8.42	4.03	15.44	7.41	3.66	3.67
	Amino sugar and nucleotide sugar	N-acetylglucosamine	**0.04**	**0.09**	**0.12**	**0.33**	**0.03**	**0.04**
Cofactors, Prosthetic Groups, Electron Carriers	Carnitine metabolism	carnitine	4.09	1.6	3.36	2.1	1.65	2.63
	Oxidative phosphorylation	methylphosphate	6.8	5.92	4.41	4.67	2.85	3.47
	Nicotinate and nicotinamide metabolism	nicotinamide adenine dinucleotide (NAD+)	8.27	5.38	9.35	2.56	4.15	1.74
	Riboflavin and FAD metabolism	riboflavin (Vitamin B2)	3.99	2.88	2.41	2.07	2.24	2.67
Lipids	Glycerolipids	1-linoleoylglycerol (1-monolinolein)	5.39	5.39	5.38	2.85	4.97	2.63
	Phospholipids	1-linoleoylglycerophosphoethanolamine*	11.64	2.86	12.46	4.13	2.61	3.52
	Phospholipids	1-linoleoylglycerophosphoinositol*	16.2	3.35	14.45	8.93	2.58	7.7
	Glycerolipids	2-linoleoylglycerol (2-monolinolein)	5.7	9.52	4.26	5.8	5.23	4.25
	Carnitine metabolism	acetylcarnitine	1.47	2.5	2.28	2.29	3.27	1.93
	Phospholipids	glycerophosphorylcholine (GPC)	1.54	1.24	1.64	2.34	1.31	2.3
	Carnitine metabolism	hydroxybutyrylcarnitine*	3.91	2.19	4.2	2.78	3.25	3.84

### Carnitine metabolism

**Figure 5:**
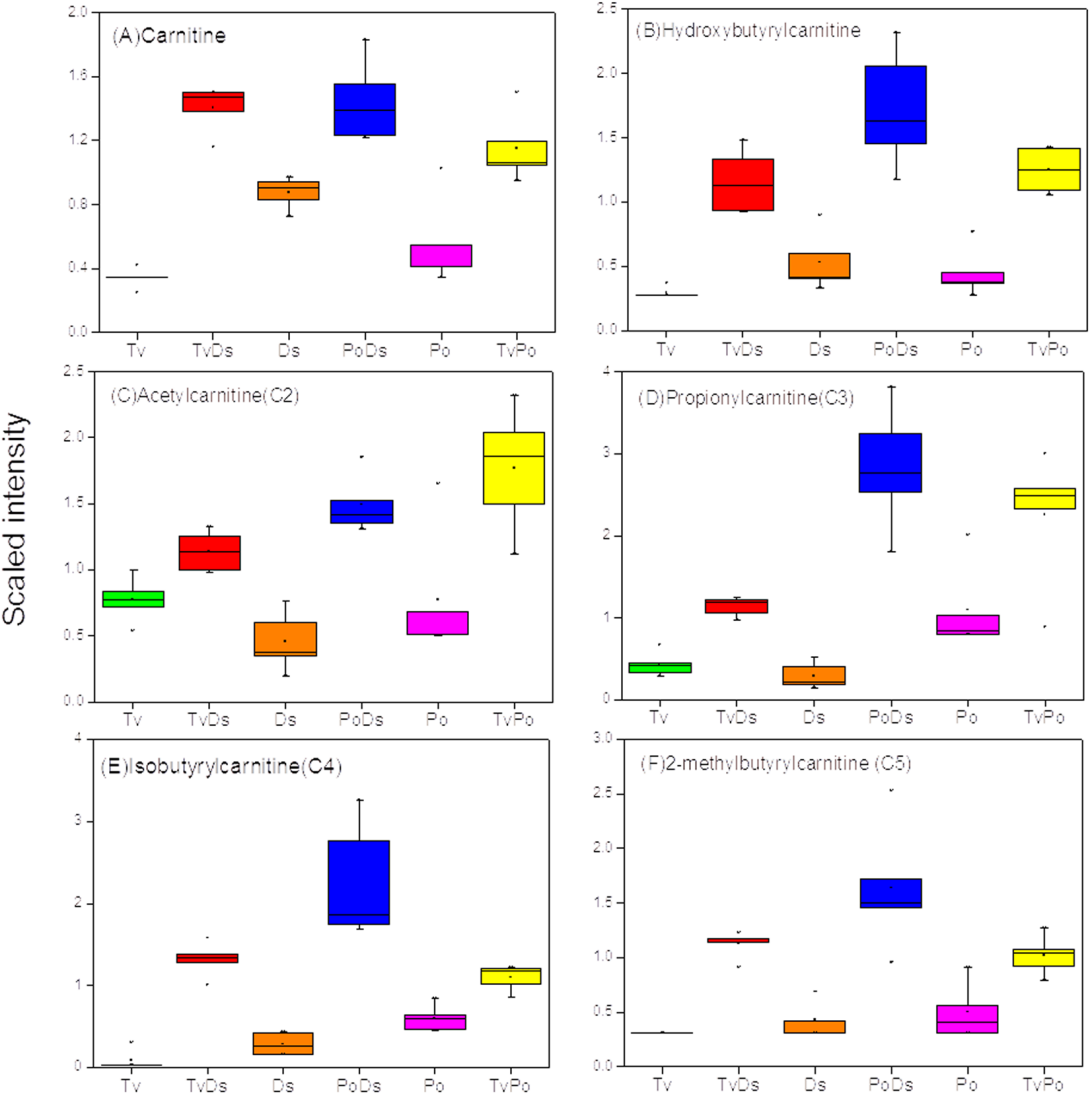
Carnitine esters significantly increase in all interaction zones. (**A**) Carnitine; (**B**) Hydroxybutyrylcarnitine; (**C**) Acetylcarnitine; (**D**) Propionylcarnitine; (**E**) isobutyrylcarnitine; (**F**) 2-methylbutyrylcarnitine.

Carnitine conjugates are derived both from the catabolism of branched chain amino acids and fatty acid oxidation^23, 24^ (Figure S2A,B). Carnitine is used to transport fatty acids across membranes, as well as serves as an alternative acyl-group receiver to CoA in order to buffer the CoA pool, typically under stressful conditions when CoA becomes limited^25^. As is shown in Fig. 5, not only the carnitine increased in all interaction zones, the catabolic products acetylcarnitine (C2), propionylcarnitine (C3), isobutyrylcarnitine (C4), 2-methylbutyrylcarnitine (C5) and hydroxybutyrylcarnitine also significantly increased in all three interactions, which reflected the cellular catabolism of amino acids and fatty acid activation. The result implied that the interaction of the three white rot fungi may trigger various catabolic processes to intensify tricarboxylic acid (TCA) cycle for energy provision.

### Lipid metabolism

**Figure 6:**
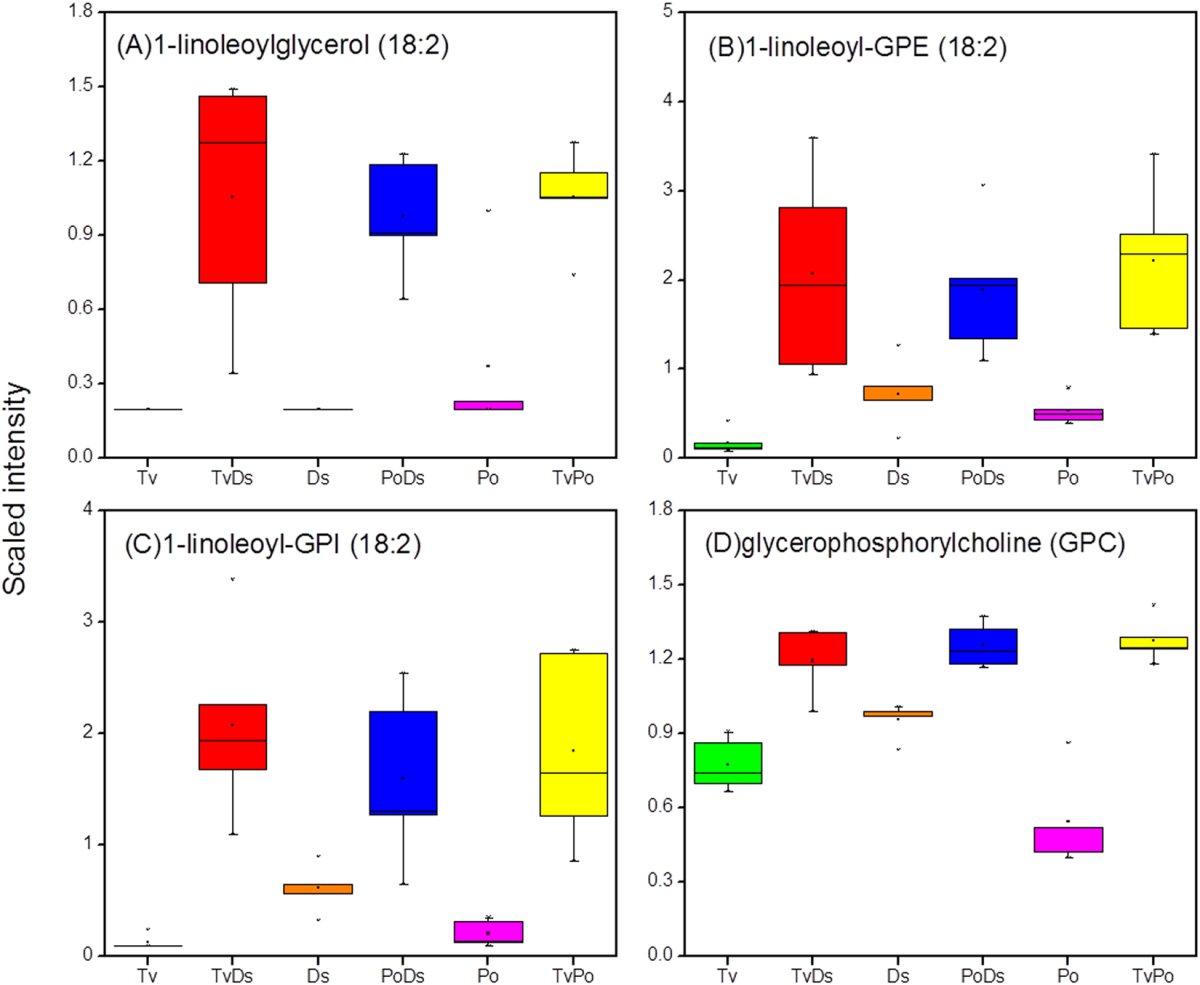
Lipolysis products significantly increase in all interaction zones. (**A**) 1-linoleoylglycerol; (**B**) 1-linoleoyl-GPE; (**C**) 1-linoleoyl-GPI; (**D**) glycerophosphocholine (GPC).

Membrane catabolism and remodeling occurs through the action of various lipases that release fatty acids from phospholipids, triacylglycerols (TAGs), diacylglycerols (DAGs) and other complex lipid^26, 27^. Although the larger complex lipids (TAGs, DAGs, cardiolipins) were not detected, many lipolysis products belonging to the group of lysolipids and monoacylglycerols significantly increased in all three interaction zones relative to each isolates. The lipolysis products included glycerophosphocholine (GPC; Fig. 6D) and its derivatives 1-linoleoylglycerol, 1-linoleoyl-GPE and 1-linoleoyl-GPI (Fig. 6A–C). GPC is generated by the degradation of phosphatidyl choline and is regarded as a very powerful osmolyte, as it is often induced under condition of osmotic stress and is served to stabilize proteins subjected to strong gradients of water potential^28^. This result suggested the existence of active lipolysis in the interaction zones of the three fungi.

### Ethylene metabolism

**Figure 7:**
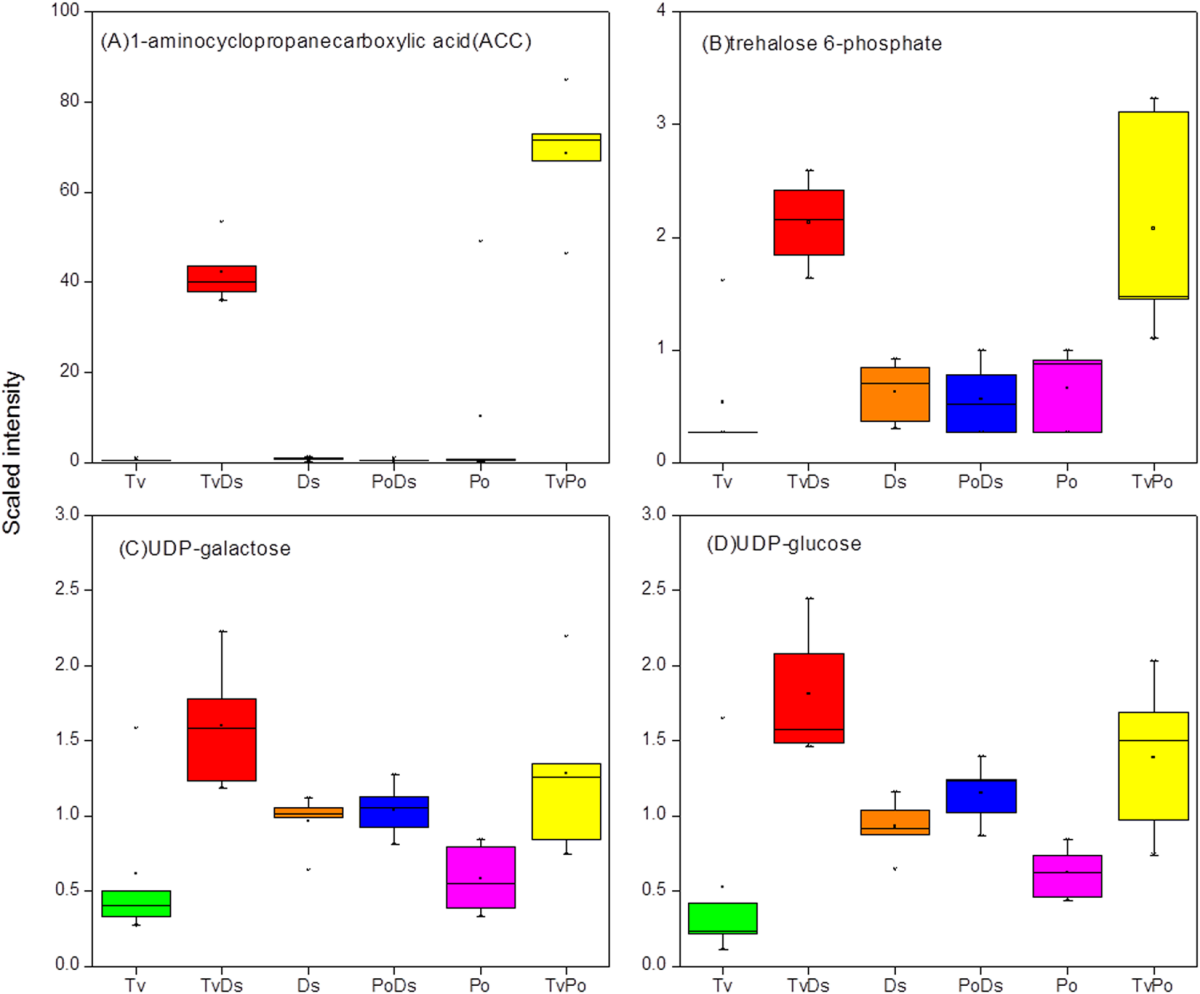
The metabolites only significantly increase in two interactions. (**A**) 1-aminocyclopropanecarbolylic acid (ACC); (**B**) Trehalose-6-phosphate, (**C**) UDP-glucose, (**D**) UDP-galactose.

There were only few compounds which exhibited opposite induction effects in the different combinations of interaction zones and isolates. One of the strongest inductions in the dataset, but only in two (TvPo and TvDs) of the three interactions, was the induction of the ethylene precursor 1-aminocyclopropanecarbolylic acid (ACC) (Fig. 7A). Ethylene is known as a stress hormone, which can be induced by a variety of stress signals, such as chemicals, metals and pathogen infection^29^. It has been reported that stress stimulation for ethylene synthesis was accompanied by the generation of reactive oxygen species (ROS) that could cause damage to cellular organelles by triggering lipid peroxidation^30, 31^. Although ethylene was not measured in this study, the high levels of its precursor ACC was detected in the TvPo and TvDs interactions, and it was significantly induced in these two interactions. Even in the TvPo interaction, the ACC was increased more than 130-fold relative to each isolates (Fig. 7A). By contrast, the accumulation of ACC was not observed in the PoDs interaction. However, the level of cyanoalanine, a by-product of ethylene synthesis that scavenges the hydrogen cyanide generated in the oxidation of ACC to form ethylene^32^, was higher in the PoDs interaction zone than in either TvPo or TvDs (Supplementary Table S3). This result suggested that ethylene may be an induced product in all interactions. However, the fact that the precursor ACC was not accumulated in PoDs revealed that there may be differential regulation mechanism of ACC oxidase in the interaction of PoDs compared with TvPo and TvDs (Figure S2D).

### Trehalose metabolism

Another pathway that was significant for only two interactions (TvDs and TvPo) was the UDP-sugars and trehalose pathway (Figure S2C). UDP-sugars like UDP-glucose and UDP-galactose, acting as the primary metabolic intermediates for the enzymatic biosynthesis of carbohydrates, were involved in cell wall biosynthesis and nucleotide sugars metabolism^33^. In addition, UDP-glucose is the direct precursor of trehalose-6-phosphate (T-6-P). T-6-P can serve to be key regulator of cell wall biosynthesis in fungi, and it was an active component that regulated the trehalose metabolic pathway^34^. Based on the antioxidant activity of trehalose, T-6-P played a key role in protecting cells from oxidative injuries, especially in cell membranes^35^. We noticed that UDP-glucose, UDP-galactose and T-6-P were synergistically increased in both TvDs and TvPo interactions (Fig. 7B–D). However, as trehalose showed decreased change in all three interactions (Supplementary Table S3), there possibly were other metabolic mechanism to degrade trehalose when cell viability was threatened under interspecific antagonism.

## Discussion

Change of metabolites *in vivo* during interactions among three white rot fungi *Trametes versicolor*, *Pleurotus ostreatus* and *Dichomitus squalens* were analysis in our study, and the metabolomic approach adopted in this study provided a broader understanding of fungal territory and resource competition during mycelial interaction by relating to the up-regulation of intracellular metabolites. There were some interesting changes on specific metabolite pathways in response to antagonistic interaction. Among the detected 279 metabolites, most of them were up-regulated in all three interactions. They were involved in the pathways of branched chain amino acids, carnitine metabolism, nicotinate and nicotinamide metabolism, glutamate family, glycerolipids, phospholipids, serine family, sucrose, glucose, fructose metabolism and TCA cycle (Figure S1). The result implied that the common metabolic processes and reactions were more active in these pathways. Importantly, except nicotinate and nicotinamide metabolism, all the other induced pathways belonged to three super pathways: carbohydrate, amino acids and lipid pathways (Supplement Table S3), indicating that a broad array of metabolic reactions with energy production could be triggered to whelm competitor in the interaction zone where fungal mycelia were overlapped.

According to the study, almost half of the compounds exhibited “synergistic” effects, which indicated they were higher or lower in the interaction zones than in either of the individual isolates. Interestingly, 17 compounds showed the same synergistic effects for all three fungal interactions, especially the most strongly induced compound tryptophan. As an aromatic amino compound, tryptophan was reported to be able to significantly induce laccase production of different fungi species^36^. In addition, many evidences revealed that laccase played an important role in a defensive response against stressful condition^37–40^ and could be significantly increased during the interaction among many white-rot fungi^8, 41–45^. Therefore, the accompanying induction of laccase was probably correlated with significant increase of tryptophan during interspecific mycelial interaction.

It has been known that a series of antagonistic responses could be triggered to compete for nutrient during combative interaction between two fungal species^1–3^. These responses were involved in intensifying fat catabolism, provision of energy substrate on account of nutrient deprivation and enhanced energy demand in competition, the generation of fatty acids (FAs), glycerols and other lysolipids which were pivotal substrates for energy production and for harmful compounds detoxification^27^. The up-regulation of lipolysis production glycerophosphocholine (GPC) and its derivatives in our work, which may contribute to stress remediation of mycelia in the interaction zone as an osmolyte^28, 46^, was associated with host’s responses. Besides, the role of carnitine in fatty acid oxidation and oxidative stress protection has been recognized in fungi^25, 47^. The generation of acetyl CoA during fatty acids β-oxidation in the peroxisome could enter mitochondria as carnitine esters for TCA metabolism^47, 48^. The function of carnitine to buffer CoA pool can also supply energy for the enhanced catabolism of fatty acids in organism^25, 48^. Moreover, carnitine also serve as a defensive molecular under stressful condition. Based on carnitine involved metabolic pathways, the synergistic up-regulation of carnitine and its conjugates in all three interactions maybe accompanied by the nutrient competition and damages of oxidative stress during fungal interspecific interactions.

We also noticed that some metabolite inductions such as ACC and T-6-P were distinct significant for one or two interactions, not for all three (Supplementary Table S4). Previous studies revealed that ethylene can be produced not only in plant but also in fungi^29, 49, 50^. As ethylene in plant was involved in the response to biotic and abiotic stresses from environmental stimuli^29, 50^, the role of ethylene in fungi might be similar to that in plant. In this study, the synergistic up-regulation of ethylene precursor ACC was detected in the interactions of TvPo and TvDs, the strongly induced ethylene pathway in at least two of the interactions possibly implied that the abiotic and oxidative stresses were stimulated in the fungal antagonistic interaction.

Trehalose is an integral compound of various glycolipids. It not only can serve as a source of energy in organism^51^, but also plays defensive role of protecting cells from oxidative damage caused by ROS, which can be overproduced during fungal combative interaction^6, 35^. UDP-sugars and T-6-P, which were two important metabolites in the widely known synthetic pathway of trehalose, were significantly increased in two interactions of TvPo and TvDs, but showed no obvious change in PoDs. The result might be caused by the differential regulation mechanisms of trehalose-6-phosphate phosphatase (T6PP) in the trehalose pathway for the various pair-wise fungal interactions^34, 52^. By contrast, trehalose was decreased in all three interaction zones, suggesting fungus probably consumed its own trehalose or glucose hydrolyzed from trehalose to supply energy and carbohydrate source when growth and nutrient limiting occurred under stressful condition^11, 52^. The similar change of trehalose has been reported in a previous study, which showed decreased level of trehalose in white rot fungus *Phanerochaete chrysosporium* under a toxic stress response^53^. This result was also probably the main reason for the significant decrease of N-acetylglucosamine in all three interaction zones (Table 1). N-acetylglucosamine is the essential component of fungal cell wall chitin^54^ and plays an vital role in regulating the intracellular signaling proteins^55^. Therefore, we supposed that the attack or defense response leaded to cell wall degradation during interspecific combative interaction, and the degradation product N-acetylglucosamine could be utilized by competitors as carbon and nitrogen source.

In summary, our metabolomics analysis revealed that intercellular metabolites differentially exist in the interspecific interaction zones relative to their isolates among three white rot fungi. We also noticed that there were common *in vivo* metabolic reactions in white rot fungi in responses to stressful condition. The intracellular metabolic changes were correlated with the interactions between different white rot fungi, which probably affected the cell wall synthesis, osmolyte production and carbon/energy regulation in the interaction zones. Importantly, this study provided important information on the understanding of defense mechanism when different fungal mycelia were physically interacted with each other. The work on identification of differential metabolites provided insight into the role of specific metabolites in the interaction zone. These results can be further used for correlation analysis with transcriptomics and proteomics to deeply understand the metabolic processes during fungal combative interaction.

## Materials and Methods

### Strains and culture

Strains of *Trametes versicolor* (Tv), *Pleurotus ostreatus* (Po) were from the Biological Resource Center, NITE (NBRC) and *Dichomitus squalens* (Ds) was from the Deutsche Sammlung von Microorganismen and Zellkulturen (DSMZ). All the three white rot fungi were cultured on potato dextrose agar (PDA) slants and stored at 4 °C. Before use, the stored fungi were inoculated onto the newly prepared PDA plates at 28 °C. Cultures were routinely cultured every 7 days.

### Mono-and paired-culture conditions

For mono-cultures, a 7 mm agar plug of a fungal pre-culture was inoculated in the center of a 90 mm petri dish containing 30 mL of Sc agar media (10 g/liter glucose,1.5/liter L-asparagine, 0.12 mg/liter Thiaminiumdichloride, 0.46 g /liter KH_2_PO_4_, 1 g/liter K_2_HPO_4_, 0.5 g/liter MgSO_4_·7 H_2_O, 2 mL trace elements, 20 g/liter Agar). The petri dishes were incubated at 28 °C for 9 days. Similarly, pair-culture experiments were inoculated with two 7 mm agar plugs from different fungal pre-culture on opposite sides of a petri dish containing Sc agar media(Fig. 1), there were three combinations(TvDs, TvPo, PoDs) in pair-culture and the petri dishes were also incubated at 28 °C for 9 days. Each mono- and pair-cultures were set five biological repeats.

### Metabolite extraction and analysis

Mycelium from the interaction zones (~2 mm wide) of three fungal pair-cultures (TvDs, TvPo, PoDs) and isolates of three mono-cultures (Tv, Po, Ds) were excised with a razor blade and then freeze dried −80 °C. Each sample received was accessioned into the Metabolon Laboratory Information Management System (LIMS) and was assigned by the LIMS a unique identifier that was associated with the original source identifier only. This identifier was used to track all sample handling, tasks, results, etc. The samples (and all derived aliquots) were tracked by the LIMS system. All portions of any sample were automatically assigned their own unique identifiers by the LIMS when a new task was created; the relationship of these samples was also tracked. All samples were maintained at −80 °C until processed. Each sample group were set five biological repeats.

Interaction and isolate samples were prepared using the automated MicroLab STAR® system from Hamilton Company to measure metabolome. A recovery standard was added prior to the first step in the extraction process for QC purposes. To remove protein, dissociate small molecules bound to protein or trapped in the precipitated protein matrix, and to recover chemically diverse metabolites, proteins were precipitated with methanol under vigorous shaking for 2 min (Glen Mills GenoGrinder 2000) followed by centrifugation. The resulting extract was divided into four fractions: one for analysis by Liquid Chromatography-Tandem Mass Spectrometry (UPLC-MS/MS) with positive ion mode electrospray ionization, one for analysis by UPLC-MS/MS with negative ion mode electrospray ionization, one for analysis by Gas Chromatography-Mass Spectroscopy (GC-MS), and one sample was reserved for backup. Samples were placed briefly on a TurboVap (Zymark) to remove the organic solvent. Each sample was then frozen and dried under vacuum, then prepared for the appropriate instrument.

### Liquid Chromatography-Tandem Mass Spectrometry (LC-MS/MS)

The LC-MS portion of the platform was based on a Waters ACQUITY ultra-performance liquid chromatography (UPLC) and a Thermo-Finnigan LTQ mass spectrometer operated at nominal mass resolution, which consisted of an electrospray ionization (ESI) source and linear ion-trap (LIT) mass analyzer. The sample extract was dried then reconstituted in acidic or basic LC-compatible solvents, each of which contained 12 or more injection standards at fixed concentrations. One aliquot was analyzed using acidic positive ion-optimized conditions and the other using basic negative ion-optimized conditions in two independent injections using separate dedicated columns (Waters UPLC BEH C18-2.1 × 100 mm, 1.7 µm). Extracts reconstituted in acidic conditions were gradient eluted using water and methanol containing 0.1% formic acid, while the basic extracts, which also used water/methanol, contained 6.5 mM ammonium bicarbonate. The MS analysis alternated between MS and data-dependent MS/MS scans using dynamic exclusion and the scan range was from 80–1000 m/z. Raw data files are archived and extracted as described below.

### Gas Chromatography-Mass Spectroscopy (GC-MS)

The samples destined for analysis by GC-MS were dried under vacuum for a minimum of 18 h prior to being derivatized under dried nitrogen using bistrimethyl-silyltrifluoroacetamide. Derivatized samples were separated on a 5% diphenyl/95% dimethyl polysiloxane fused silica column (20 m × 0.18 mm ID; 0.18 um film thickness) with helium as carrier gas and a temperature ramp from 64° to 340 °C in a 17.5 min period. Samples were analyzed on a Thermo-Finnigan Trace DSQ fast-scanning single-quadrupole mass spectrometer using electron impact ionization (EI) and operated at unit mass resolving power. The scan range was from 50–750 m/z. Raw data files are archived and extracted as described below.

### Data extraction and compound identification

Raw data was extracted, peak-identified and QC processed using Metabolon’s hardware and software. Compounds were identified by comparison to library entries of purified standards or recurrent unknown entities. Metabolon maintains a library based on authenticated standards that contains the retention time/index (RI), mass to charge ratio (m/z), and chromatographic data (including MS/MS spectral data) on all molecules present in the library. Furthermore, biochemical identifications are based on three criteria: retention index within a narrow RI window of the proposed identification, accurate mass match to the library +/−0.005 amu, and the MS/MS forward and reverse scores between the experimental data and authentic standards. The MS/MS scores are based on a comparison of the ions present in the experimental spectrum to the ions present in the library spectrum. While there may be similarities between these molecules based on one of these factors, the use of all three data points can be utilized to distinguish and differential metabolites. More than 3300 commercially available purified standard compounds have been acquired and registered into LIMS for distribution to both the LC-MS and GC-MS platforms for determination of their analytical characteristics. Additional mass spectral entries have been created for structurally unnamed metabolites, which have been identified by virtue of their recurrent nature (both chromatographic and mass spectral).These compounds have the potential to be identified by future acquisition of a matching purified standard or by classical structural analysis.

### Data analysis

Data were normalized for internal consistency by processing a constant weight per volume of extraction solvent for each sample. Data were scaled to the median value for each compound, then missing values were imputed with the minimum detected value for that compound (both RAW and SCALED IMPUTED data tables are provided in Supplementary Table S1 and S2). Moreover, a variety of curation procedures were carried out to ensure that a high quality data set was made available for statistical analysis and data interpretation. The QC and curation processes were designed to ensure accurate and consistent identification of true chemical entities, and to remove those representing system artifacts, mis-assignments, and background noise. Metabolon data analysts use proprietary visualization and interpretation software to confirm the consistency of peak identification among the various samples. Library matches for each compound were checked for each sample and corrected if necessary.

## Electronic supplementary material

Supplementary Figures

Supplementary Tables
