# Supplementary material for: Metabolomic differential analysis of interspecific interactions among white rot fungi Trametes versicolor, Dichomitus squalens and Pleurotus ostreatus
Source: Sci Rep. 2017 Jul 13;7:5265. doi: 10.1038/s41598-017-05669-3 (PMC5509712; doi:10.1038/s41598-017-05669-3)
Supplement: Supplementary file 1 — Supplementary Figures [file 41598_2017_5669_MOESM1_ESM.pdf]

Metabolomic differential analysis of interspecific interactions among white rot fungi *Trametes versicolor*, *Dichomitus squalens* and *Pleurotus ostreatus*

Feng Luo[1], Zixuan Zhong[1], Li Liu[1] , Yasuo Igarashi, Deti Xie , Nannan Li[*]

Research Center of Bioenergy and Bioremediation, College of Resources and Environment, Southwest University, Beibei, Chongqing 400715, People's Republic of China

[1] These authors contributed equally to this work

[*]Corresponding author: Nannan Li (NL) linan029@163.com

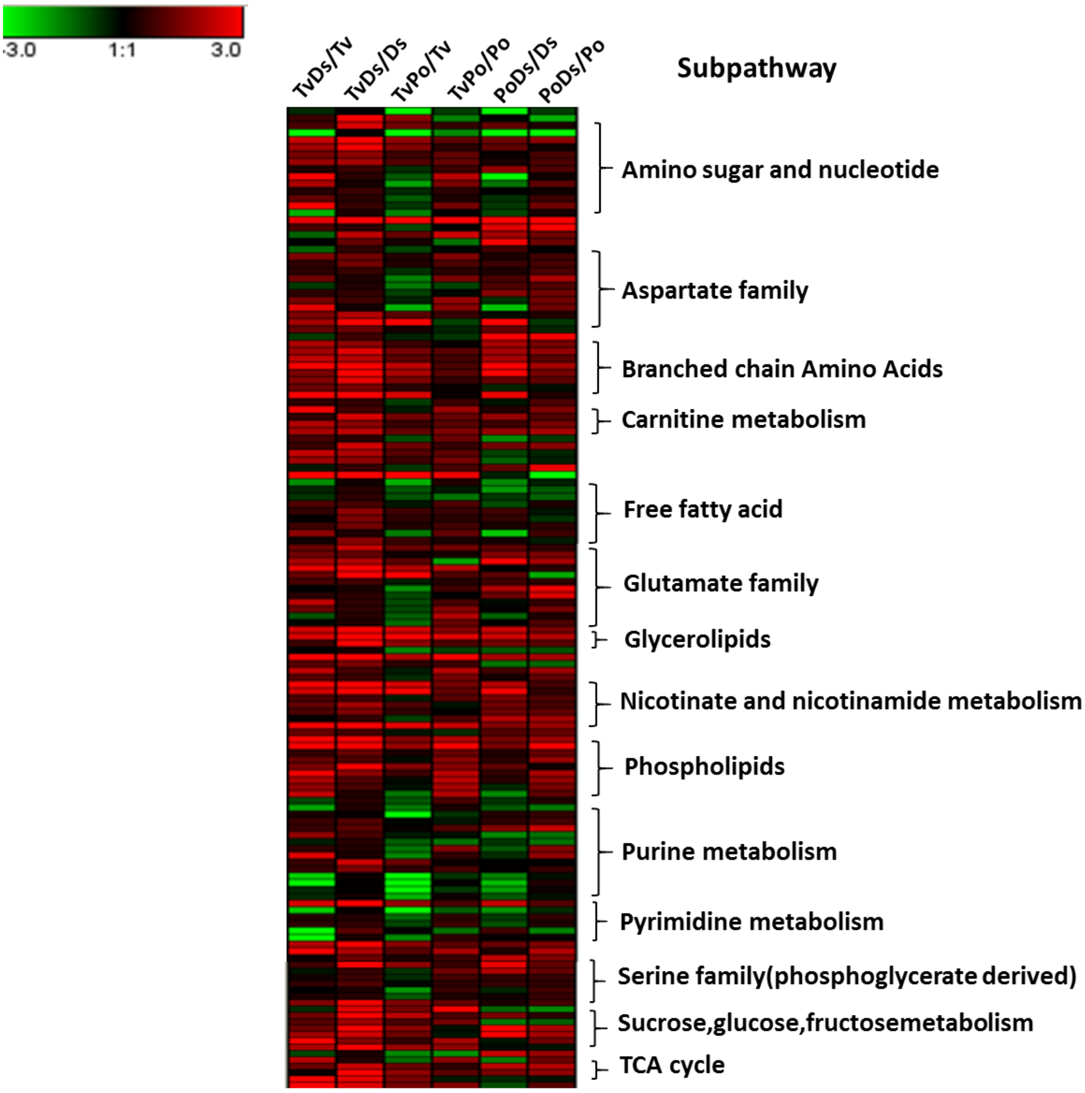

Figure S1. Heat map of fold change value of all detected metabolite contents from five biological replicates. The metabolomic data include all detected 279 metabolites. Red and green indicate increased and decreased metabolites in the interaction zones compared to both isolates. Each fold change value was calculated in log_2.

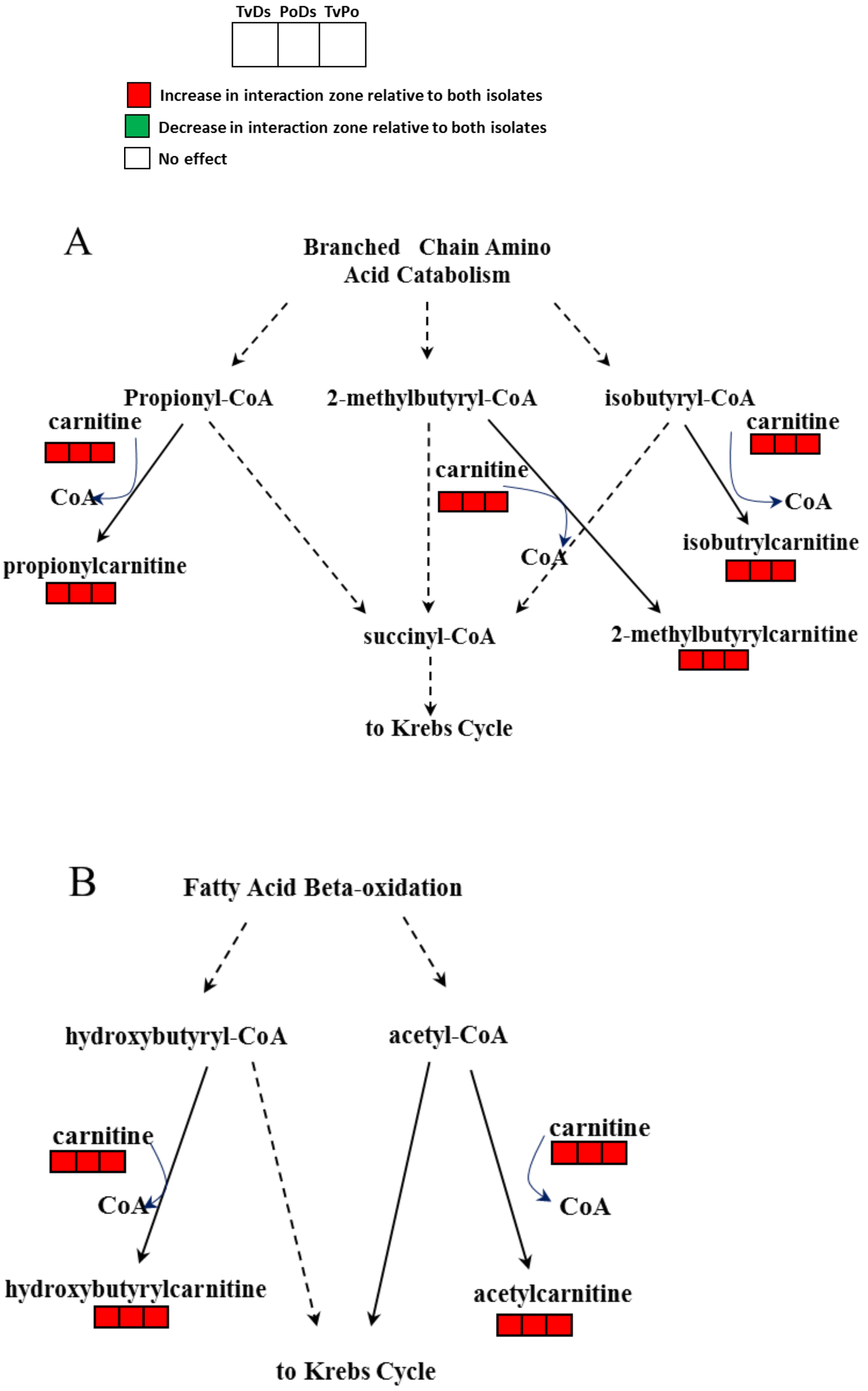

TvDs PoDs TvPo
Increase in interaction zone relative to both isolates
Decrease in interaction zone relative to both isolates
No effect
A
Branched Chain Amino Acid Catabolism
Propionyl-CoA
2-methylbutyryl-CoA
isobutyryl-CoA
carnitine
CoA
propionylcarnitine
carnitine
CoA
carnitine
CoA
isobutrylcarnitine
succinyl-CoA
2-methylbutyrylcarnitine
to Krebs Cycle
B
Fatty Acid Beta-oxidation
hydroxybutyryl-CoA
acetyl-CoA
carnitine
CoA
carnitine
CoA
hydroxybutyrylcarnitine
acetylcarnitine
to Krebs Cycle

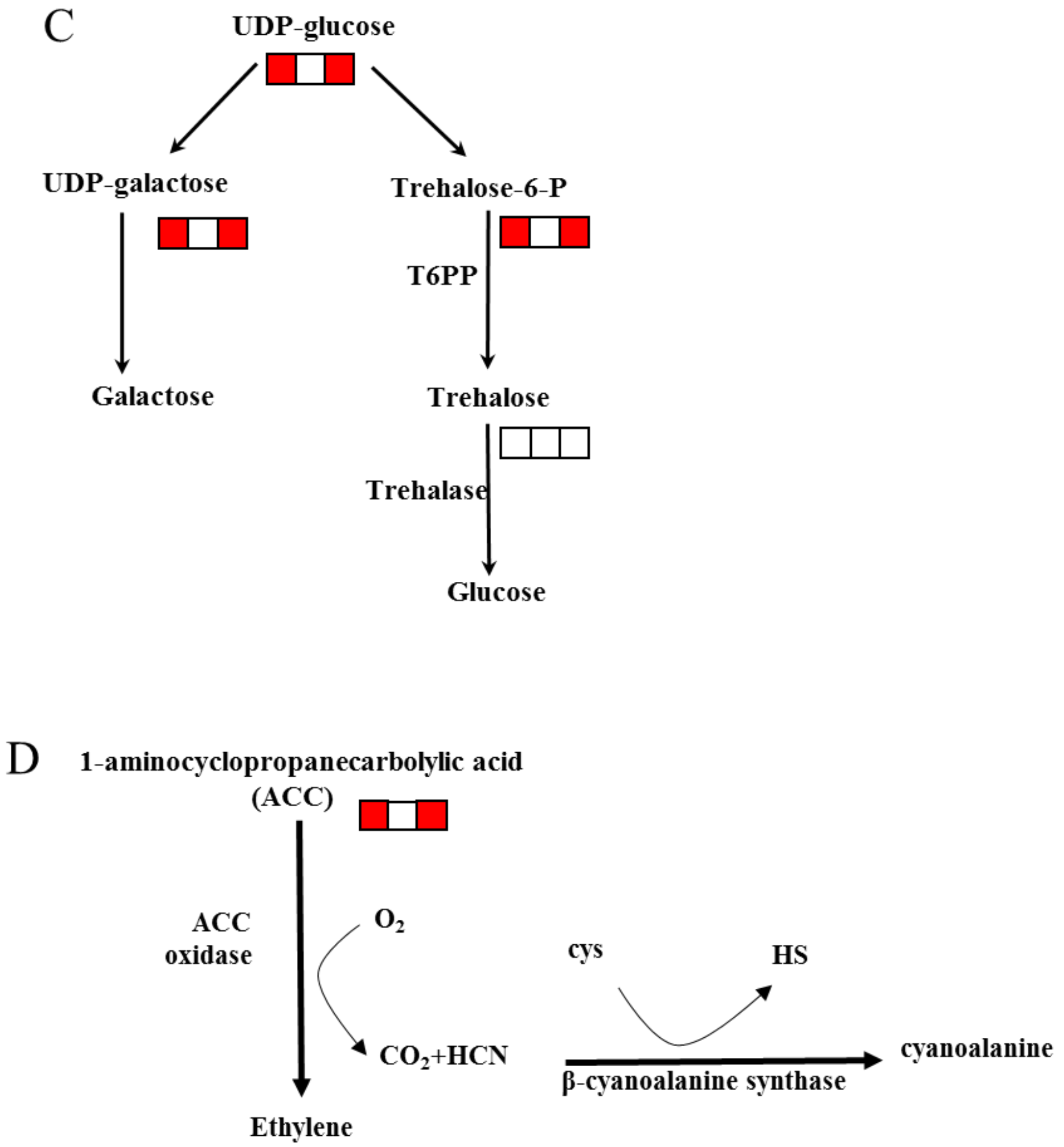

Figure S2. The significantly induced metabolic pathway in vivo. (A) Branched chain amino acids catabolic pathway; (B) Fatty acid oxidation pathway; (C) UDP-glucose catabolic pathway; (D) Ethylene synthesis pathway.
